# Early Childhood Caries and Its Associated Factors Among 5-Year-Old Children in Shenzhen City, China: A Cross-Sectional Study

**DOI:** 10.3390/dj13120552

**Published:** 2025-11-24

**Authors:** Anthony Yihong Cheng, Jieyi Chen, Faith Miaomiao Zheng, Duangporn Duangthip, Chun Hung Chu

**Affiliations:** 1 Faculty of Dentistry, The University of Hong Kong, Hong Kong SAR, China; ayhcheng@connect.hku.hk (A.Y.C.); zhengmm@connect.hku.hk (F.M.Z.); 2 Guanghua School of Stomatology, Hospital of Stomatology, Sun Yat-sen University, Guangzhou 510055, China; 3 Division of Pediatric Dentistry, The Ohio State University, Columbus, OH 43210, USA; duangthip.2@osu.edu

**Keywords:** early childhood caries, prevalence, risk factors, preschool children, China

## Abstract

**Background:** Early childhood caries (ECC) remains a critical public health challenge, yet recent prevalence data and risk factors are scarce in rapidly urbanizing regions like Shenzhen City, China. **Objectives**: This study aimed to assess ECC prevalence and identify risk factors among 5-year-old children in Shenzhen City. **Methods**: This cross-sectional survey was conducted in Shenzhen City in 2024, recruiting 5-year-old children through multistage sampling from kindergartens. Self-administered parental questionnaires were distributed to collect data such as demographic characteristics, socioeconomic background and oral health-related behaviors. One trained dentist conducted the oral examination in kindergartens using ball-ended community periodontal index probes and disposable dental mirrors with an intra-oral light-emitting diode light attached. Dental caries was assessed using diagnosis criteria recommended by World Health Organization. The decayed, missing, and filled primary teeth (dmft) were recorded. Zero-inflated negative binomial regression was applied to identify associations between risk factors and ECC. **Results**: Among 1462 participants (86% response rate), ECC prevalence was 58% (mean dmft: 2.5 ± 3.4), with untreated decay (dt) accounting for 92% of cases. Socioeconomic factors, including low family income (*p* < 0.001), non-local residency (*p* < 0.001), and low caregiver education level (*p* = 0.012), were significantly associated with higher dmft scores. Behavioral factors such as frequent sugary drink consumption (*p* = 0.005), lack of parental brushing assistance (*p* = 0.027), and non-fluoride toothpaste use (*p* = 0.008) also contributed to the risk of ECC. **Conclusions**: Over half of Shenzhen City’s 5-year-olds suffered from ECC, predominantly untreated, driven by socioeconomic disparities and modifiable behavioral factors. Public health strategies must prioritize parental education, fluoride use and early preventive practices to reduce the burden of ECC.

## 1. Introduction

Early childhood caries (ECC) is defined as the presence of one or more decayed, missing, or filled tooth surfaces in any primary tooth among children under 6 years of age [1]. As one of the most common chronic diseases affecting young children globally, ECC affects approximately 514 million children worldwide, representing a significant public health challenge [2,3]. The prevalence of ECC varies considerably across regions, with developing countries bearing a disproportionate burden. Globally, dental caries prevalence in primary teeth is estimated at 48%, while Asia reports higher rates than Europe and Latin America [4,5]. In Southeast Asia, some countries report prevalence rates approaching 90% among preschool children [6].

China faces substantial ECC challenges, with the Fourth National Oral Health Epidemiological Survey (2017) revealing that 72% of 5-year-old children have dental caries experience [7]. Significant regional disparities exist within China, with Guangdong Province ranking among the top ten highest prevalence rates in 31 mainland provinces, paradoxically inconsistent with its advanced economic development [8]. A provincial survey in Guangdong during 2015 to 2016 found 68% 3- to 5-year-old children had ECC, with a mean dmft score of 4.4, while the filled rate was only 1.2% [9]. Recent longitudinal analysis indicates that education- and economy-related inequalities in ECC decreased between 2005–2015 but increased again by 2021, particularly in rural areas [10].

Shenzhen was established as China’s first Special Economic Zone in 1980 [11]. It has transformed from a fishing village into Guangdong Province’s second most populous city, with over 13 million residents. This rapid development created unique characteristics, including diverse demographics, substantial income disparities, and the youngest population structure among China’s megacities [12]. Shenzhen’s economic prosperity has significantly influenced dietary patterns relevant to oral health, including high consumption of processed foods and sugar-sweetened beverages [13]. Additionally, predominance of dual-income families with extended working hours may impact parental supervision of children’s oral health behaviors. These unique demographic, economic, and social characteristics make Shenzhen particularly suitable for investigating ECC patterns and determinants in China’s economically advanced cities.

ECC is multifactorial, influenced by biological, behavioral and social determinants such as sugar consumption, oral hygiene, toothbrushing initiation, fluoride exposure, and socioeconomic background [14,15]. These risk factors contribute to the development of ECC through different biological pathways [16]. Poor dietary habits provide substrate for cariogenic bacteria. These bacteria produce acids that demineralize the enamel. Inadequate oral hygiene allows plaque to build up, leading to longer exposure to acids. Limited access to fluoride reduces the enamel’s ability for remineralization. The World Health Organization (WHO) recommends epidemiological surveys at five-year intervals to monitor oral health status [17]. Despite Shenzhen’s economic development and strategic importance, limited recent data exist regarding ECC prevalence and risk factors among Shenzhen’s preschool population. In contrast, recent studies using WHO criteria in other major Chinese cities report varying ECC prevalence among 5-year-old children—Hong Kong (42%), Shanghai (51%), and Guangzhou (65%)—highlighting significant urban variations [18,19,20]. Understanding ECC patterns in Shenzhen is essential not only for local public health planning but also for informing oral health strategies in other rapidly developing Chinese cities facing similar demographic and socioeconomic transitions.

This study aimed to assess ECC prevalence and severity among 5-year-old children in Shenzhen City and identify associated sociodemographic, behavioral, and family-related factors. Results of this study may provide evidence-based recommendations for public health interventions in rapidly urbanizing populations.

## 2. Methods

This cross-sectional study was conducted in 2024 with ethical approval from the Institutional Review Board of the University of Hong Kong/Hospital Authority Hong Kong West Cluster (IRB: UW 23-064, 22 February 2023) and the Medical Ethics Committee of Shenzhen Center for Disease Control and Prevention (IRB: QS2023020059, 16 March 2023). The study followed the Declaration of Helsinki principles, and written informed consent was obtained from the parent of each child prior to participation. The study is reported in accordance with the STrengthening the Reporting of OBservational studies in Epidemiology (STROBE) guidelines (Table S1) [21].

### 2.1. Sample Size Calculation and Sample Selection

The study was conducted among 5-year-old children in Shenzhen City, Guangdong Province, China. The sample size estimation was based on previous caries prevalence data (approximately 50%) [22]. The confidence interval was set at 5% (CI: 45–55%) with a 95% confidence level, yielding a required sample size of 384 children. With an estimated response rate of 80%, the minimum number of children to be invited was 480 per district. Given the two-district design, the initial target was 960 children. To maximize statistical power for detecting risk factors, recruitment continued until all eligible children from selected kindergartens were included.

Futian and Nanshan Districts were selected using a non-probabilistic approach which can represent Shenzhen City’s urban core areas. These two districts were well-developed and densely populated areas of Shenzhen City, housing central business districts and major residential communities. Both districts have well-established educational infrastructure and diverse socioeconomic populations, making them representative of Shenzhen’s urban demographic characteristics. Samples were then selected using the multistage sampling method. The ratio of children invited was determined according to the population distribution between clusters. Registered kindergartens in each district were numbered sequentially and selected by simple random sampling using computer-generated random numbers. The inclusion criterion was generally healthy children. Children who were uncooperative, had special needs, underwent prolonged medication use, or had severe chronic diseases were excluded.

### 2.2. Questionnaire Survey

Self-administered parental questionnaires and consent forms were distributed to the parents or legal guardians of children in the selected kindergartens. Parents or legal guardians completed questionnaires without assistance and submitted them before the day of clinical examination. The questionnaire consisted of three sections: (i) the child’s demographic background including sex, age, household registration type, only-child or multi-child family status and primary caregiver; (ii) socioeconomic status including family monthly income and parents’ education level; (iii) oral health-related behaviors including snacking habits, pre-sleep eating habits, feeding type in the first 6 months of life, toothbrushing habits and dental visit experience. The questionnaire was adapted from validated questionnaires used in our previous ECC studies [23,24,25] and modified for the local Chinese context. Content validity was established through expert panel review. Pilot testing was conducted with 30 parents from non-participating kindergartens, with minor language modifications made based on feedback. Kindergarten teachers assisted with the collection of consent forms and questionnaires from all eligible families. Returned questionnaires with missing or incomplete answers were followed up by telephone.

### 2.3. Clinical Examination

A single calibrated examiner (AYC) conducted all clinical assessments under the training and supervision of experienced dental epidemiologists (CHC). The examiner underwent training in caries diagnosis using visual–tactile assessment according to WHO diagnostic criteria [17]. Calibration in caries diagnosis was performed by an experienced examiner (JC) and the examiner (AYC). Approximately 10% of participating children underwent random re-examination on the same day to evaluate intra-examiner reliability. The study children were positioned supine on small tables in the kindergartens. Clinical examinations were conducted using a ball-ended WHO Community Periodontal Index (CPI) probe and a disposable dental mirror with an intra-oral light-emitting diode (LED) light attached. Decayed teeth (dt) were recorded when dentine lesions presented unmistakable cavitation or when carious lesions coexisted with restorations. Missing teeth (mt) were recorded when extraction resulted from caries. Filled teeth (ft) were recorded when permanent restorations without caries were present. Radiographic examination was not performed. Following each child’s oral examination, an oral health report was sent to the parent.

### 2.4. Statistical Analysis

Data were entered using Microsoft Office Excel 2019, with two field assistants performing double-checking to prevent mis-entry. Statistical analyses were conducted using SPSS version 27.0 (SPSS Inc., Chicago, IL, USA) and STATA version 18 (StataCorp, College Station, TX, USA). Children with missing data were excluded from analyses. Descriptive statistics were presented as frequencies and percentages. Intra-examiner agreement was evaluated using Kappa statistics. Chi-square tests assessed differences in risk factors between caries and caries-free groups, while Mann–Whitney U tests examined dmft score distributions across variables. Independent variables with *p*-values < 0.10 from univariate analyses were included as covariates in regression models. Zero-inflated negative binomial (ZINB) regression was selected for analyzing factors associated with ECC due to the high proportion of children with no caries experience and the over-dispersed count nature of dmft scores. Backward stepwise selection removed non-significant variables, retaining only those with *p*-values < 0.05. The level of significance for all tests was set at 0.05.

## 3. Results

A total of 1710 5-year-old children from 27 kindergartens were invited to participate in the study. Of these, 1477 children with parental consents completed the clinical examination, resulting in a response rate of 86% (1477/1710). Intra-examiner reliability was assessed through re-examination of approximately 10% of the children, yielding a Kappa coefficient of 0.95, indicating excellent agreement between examinations. Among the 1477 children who participated, 15 children did not complete the questionnaire, yielding 1462 children with complete data for analysis. All descriptive data and statistical analyses were weighted to account for demographic differences.

**Figure 1:**
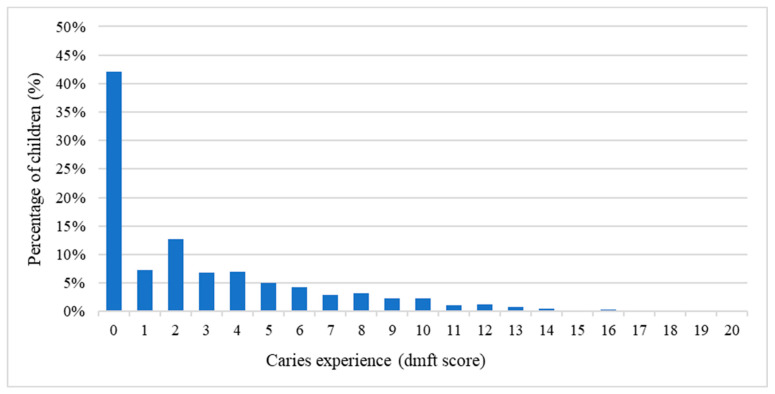
Caries experience in dmft scores of the 5-year-old children (*n* = 1462).

The study population comprised 766 boys (52%) and 696 girls (48%). The overall caries prevalence among 5-year-old children in Shenzhen City was 58% (Table 1). Boys exhibited a slightly higher caries prevalence (59%) compared to girls (56%), though this difference was not statistically significant (*p* = 0.254). Children with non-local residency showed higher caries prevalence (59%) than those with local residency (57%), but this difference was also not statistically significant (*p* = 0.537). The mean dmft score was 2.8 ± 3.5, with the untreated decayed teeth (dt) accounting for the majority of the index (2.5 ± 3.4). The missing teeth (mt) was 0.01 ± 0.12, while the filled (ft) was 0.20 ± 0.84. No significant difference in dmft scores was observed between boys and girls (*p* = 0.306). However, children with non-local residency had higher dmft scores (3.2 ± 4.0) compared to those with local residency (2.6 ± 3.3), with a trend toward significance (*p* = 0.062). The frequency distribution of dmft scores was positively skewed, with a skewness of 1.5, shown in Figure 1. Caries experience prevalence was higher in maxillary than mandibular teeth. Maxillary central incisors demonstrated the highest caries prevalence (34%), followed by mandibular molars (24%) and maxillary molars (17%), while mandibular lateral incisors had the lowest prevalence (1.3%). The distribution of the tooth position of ECC is shown in Figure S1.

Several sociodemographic and behavioral factors were significantly associated with caries prevalence in bivariate analysis (Table 2). Most children (62%) had siblings, and they demonstrated higher caries prevalence (61%) compared to only children (52%, *p* < 0.001). Nearly half of the families (47%) had monthly income below the median (≤20,000 RMB), which was associated with increased caries prevalence (63% vs. 53%, *p* < 0.001). Children whose caregivers had education below tertiary level (48%) showed higher caries prevalence (63%) than those with tertiary-educated caregivers (53%, *p* < 0.001).

The vast majority of children (89%) were breastfed in the first 6 months, while non-breastfed children (11%) exhibited higher caries prevalence (65% vs. 57%, *p* = 0.046). Most children (65%) had the habit of snacking before bed, which was associated with increased caries prevalence (60% vs. 53%, *p* = 0.013). Children who consumed sugary drinks at least twice daily (21%) had significantly higher caries prevalence (65% vs. 56%, *p* = 0.004). Most children (66%) started brushing before age 2, while those who started brushing at age 2 or later had higher caries rates (65% vs. 54%, *p* < 0.001).

Many children (59%) lacked parental assistance in brushing, which was associated with increased caries prevalence (61% vs. 53%, *p* = 0.001). Use of fluoride toothpaste (59%) showed a marginally significant protective effect (57% vs. 66%, *p* = 0.051). Children with dental visit experience (48%) showed higher caries prevalence (68% vs. 48%, *p* < 0.001), likely reflecting treatment-seeking behavior rather than preventive care.

In the Mann–Whitney *U*-test, significantly higher median dmft scores were observed among children who had siblings, whose caregivers had education below tertiary level, who snacked before bed, who consumed sugary drinks at least twice daily, who had no brushing below aged 2, who had no assistance in brushing, who had no use of fluoride toothpaste, and who had dental visit experience (Table 3). Family income (*p* = 0.092) and breastfeeding in the first 6 months (*p* = 0.065) showed a trend toward significance, while all other factors demonstrated significant associations (all *p* < 0.05).

According to Vuong’s test, the zero-inflated negative binomial (ZINB) model provided a better fit than the Poisson distribution (*p* < 0.001). The results from the final model of the zero-inflated part (dmft = 0) indicated that six variables were significantly associated with the chance of having ‘no caries experience’ (dmft = 0). Children who were only children (OR = 1.48), came from families with income above the median (OR = 1.92), consumed sugary drinks less than twice daily (OR = 1.66), received parental assistance in brushing (OR = 0.73), used fluoride toothpaste (OR = 0.68), and had never visited a dentist (OR = 2.84), had an increasing probability of having ‘no caries experience’ (*p* < 0.05) (Table 4). In addition, the results of the final ZINB regression model (dmft > 0) revealed that six factors were statistically significantly associated with the mean dmft score in the negative binomial part. Children from families with income below the median (IRR = 0.77), with non-local residency (IRR = 1.35), whose caregivers had education below tertiary level (IRR = 0.85), who snacked before bed (IRR = 0.83), who started brushing at age 2 or later (IRR = 1.14), and who had dental visit experience (IRR = 0.79), had significantly higher dmft scores (*p* < 0.05) (Table 5).

## 4. Discussion

This cross-sectional study revealed that over half of the recruited 5-year-old children in Shenzhen City have experienced dental caries, with each child averaging nearly three affected teeth. Notably, Shenzhen’s ECC prevalence falls below China’s national average, where the latest National Oral Health Survey reported 72% prevalence among 5-year-olds [7]. This finding positions Shenzhen favorably within the national context, suggesting that urban development and healthcare infrastructure may contribute to better oral health outcomes compared to less developed regions. Nevertheless, sustained efforts remain imperative to improve the situation and address oral-health challenges in Shenzhen.

A major oral-health challenge confronting Shenzhen City is the high level of unmet treatment needs. This situation is particularly concerning given Shenzhen’s status as one of China’s most economically developed cities. The factors contributing to this paradox of high unmet treatment needs despite economic prosperity can be analyzed across three dimensions. At the individual level, children themselves may face challenges to receiving dental care. Many children experience dental anxiety and fear of dental procedures, which can lead to avoidance behaviors and resistance to treatment [26]. Additionally, young children may have limited understanding of oral health importance and may not communicate dental pain or discomfort effectively to their caregivers, resulting in delayed recognition of treatment needs [27]. At the family level, two critical barriers emerge. First, there exists a significant knowledge gap among parents or caregivers regarding the importance of primary teeth and early dental intervention [28]. Many parents still hold the misconception that primary teeth are temporary and therefore do not require treatment, leading to delayed or avoided dental care [29]. Second, healthcare policy gaps exist where dental care, particularly for children, receives insufficient coverage under public health insurance schemes, imposing economic burdens on low-income migrant families [30,31]. At the community level, systemic healthcare challenges persist. The healthcare system structure in Shenzhen prioritizes curative over preventive care, with limited integration of oral health services into routine pediatric healthcare visits [32,33]. This fragmented approach means that early caries detection and intervention opportunities are frequently missed. Additionally, despite economic development, access barriers persist due to uneven distribution of pediatric dental specialists across the city, with most concentrated in central urban areas while underserved communities in peripheral districts face geographic and logistical challenges [34]. These multi-level systemic issues collectively explain why Shenzhen, despite its economic advantages, continues to struggle with high levels of untreated dental caries, highlighting the need for comprehensive policy reforms that address barriers at individual, family, and community levels.

Socioeconomic inequalities remain a persistent challenge in Shenzhen’s oral health landscape. This study found that children from families with higher education levels and greater financial resources demonstrating significantly lower caries prevalence. This finding aligns with recent research in Guangdong Province demonstrating persistent socioeconomic inequalities in ECC [8]. Parents with higher education levels typically possess better oral health knowledge, more favorable attitudes toward preventive care, and greater ability to navigate healthcare systems effectively [35,36]. Another family-level risk factor was family size. Children from multi-child family were found to have higher caries risk. The association between having siblings and increased caries risk may reflect resource dilution within families, where parental attention, time, and financial resources are distributed among multiple children [37]. Recent international research supports this finding, demonstrating that families with fewer children are associated with significantly lower odds of dental caries, as children from smaller families exhibit better oral health practices, including more regular brushing and dental visits [38,39]. This finding is particularly relevant in the Chinese context, where the relaxation of the one-child policy has increased the number of multi-child families, potentially creating new oral health challenges [40]. To address these challenges, targeted interventions are needed, including family-centered oral health education programs, resource optimization strategies for larger families, policy reforms that account for multiple children in healthcare coverage, and community support programs specifically designed for multi-child families [41].

Another challenge is the prevalent deleterious oral habits among children in Shenzhen City. Deleterious feeding practices, dietary habits, and oral hygiene behaviors were all well-established caries risk factors. The association between frequent sugary drink consumption and higher caries prevalence supports the well-established dose–response relationship between sugar consumption frequency and caries development [42]. The habit of bedtime snacking represents a modifiable risk factor, as nighttime eating prolongs exposure of teeth to cariogenic substrates when salivary flow is naturally reduced [43,44]. Children and parents in Shenzhen should be instructed to reduce the consumption of snacks and sugary drinks. However, parental assistance in toothbrushing should be advocated. It was reported that parental assistance in toothbrushing was associated with lower caries prevalence, which underscores the critical importance of parental involvement in young children’s oral care, as children under 6 years typically lack the manual dexterity and cognitive development necessary for effective independent brushing [45,46]. Research consistently demonstrates that parental supervision and assistance should continue until children develop adequate fine motor skills, typically around 8–10 years of age [47]. Conversely, the counterintuitive finding that children with dental visit experience showed higher caries prevalence reveals a treatment-oriented rather than prevention-focused healthcare approach. This suggests that dental consultations are primarily reactive, occurring after caries development rather than as preventive measures.

This study also found that the adoption of fluoride agents among Shenzhen children was low. Only 59% of children used fluoride toothpaste, representing a critical gap in preventive care that may partly explain the persistent caries burden despite Shenzhen’s economic prosperity. The protective effect of fluoride toothpaste use supports extensive evidence from systematic reviews demonstrating the caries-preventive benefits of fluoride [48]. This finding is particularly striking given that fluoride toothpaste is readily available and affordable in the city. The underutilization may stem from parental concerns about fluoride safety, especially regarding ingestion by young children, lack of awareness about fluoride’s caries-preventive benefits, cultural preferences for traditional oral care products, and insufficient guidance from healthcare providers. The comparison with neighboring Hong Kong provides valuable insights for potential interventions. Hong Kong, sharing similar urban environments and socioeconomic development levels with Shenzhen, has achieved remarkable success in ECC reduction—from 51% prevalence in 2011 to 42% in 2021 [18]. This improvement resulted from comprehensive fluoride strategies including water fluoridation, school-based supervised toothbrushing programs, and innovative silver diamine fluoride applications with arrest rates of 65–91% for cavitated lesions [49]. Our findings suggest that targeted educational interventions to improve fluoride toothpaste utilization, coupled with enhanced healthcare provider training on fluoride counselling, could yield meaningful improvements in caries prevention among Shenzhen’s preschool population.

This study provides several important contributions to the understanding of ECC in rapidly developing Chinese cities. The multistage sampling approach successfully captured representative data from Shenzhen’s urban core, with Futian and Nanshan Districts providing comprehensive coverage of diverse socioeconomic populations. These districts were strategically chosen as they represent the most developed and densely populated areas of Shenzhen, housing the city’s central business districts and major residential communities with well-established educational infrastructure. The robust sample size enables confident extrapolation to the broader 5-year-old population in Shenzhen’s developed urban areas. The findings provide important baseline data for developing targeted interventions in Shenzhen and potentially other Chinese cities. The identification of specific risk factors enables the development of targeted prevention strategies, including kindergarten-based programs that could reach all children regardless of family circumstances [50]. Such programs could include daily supervised toothbrushing, oral health education for children and parents, and regular dental screenings [51]. Given Shenzhen’s proximity to Hong Kong’s successful initiatives, these experiences could inform comprehensive prevention strategies addressing interventions across all socioeconomic strata.

Several limitations should be acknowledged when interpreting these findings. First, the non-probabilistic convenience sampling from only two districts may limit the generalizability of findings to all children in Shenzhen or other urban areas in China. While the selected districts represent diverse socioeconomic backgrounds, the results may not be fully representative of the entire city’s population. However, the samples were selected using the probabilistic approach to maintain representativeness. Second, the cross-sectional design precludes causal inferences about risk factor relationships. Third, the study relied on parental consent and questionnaire return, which may exclude lower-educated or less health-aware families, introducing participation bias. To address this limitation, Kindergarten teachers assisted with the collection of consent forms and questionnaires from all eligible families. Additionally, in accordance with WHO recommendations, diagnoses were conducted at the tooth level, thereby limiting a more detailed analysis due to the lack of surface-specific information. For ethical reasons, no radiographic examination was performed to minimise the risk of radiation on young children.

## 5. Conclusions

More than half of Shenzhen’s 5-year-olds had untreated ECC, linked to socioeconomic and behavioural factors. Strengthening parental education, fluoride use, and early prevention should be public-health priorities to reduce the burden of ECC.

## Figures and Tables

**Table 1:** Dental caries prevalence (%) and experience (dmft) of the 5-year-old children (n = 1462).

Independent Variables (*n*)	Caries Prevalence (*n*)	*p*-Value ^1^	dmft Mean ± SD	dt Mean ± SD	mt Mean ± SD	ft Mean ± SD	Rank of Median dmft Score	*p*-Value ^2^
All children (1462)	58% (846)		2.76 ± 3.53	2.54 ± 3.37	0.01 ± 0.12	0.20 ± 0.84		
Sex		0.254						0.306
Boys (766)	59% (454)		2.85 ± 3.62	2.64 ± 3.34	0.01 ± 0.14	0.20 ± 0.82	742	
Girls (696)	56% (392)		2.65 ± 3.42	2.44 ± 3.29	<0.01	0.21 ± 0.85	720	
Residency		0.537						0.062
Local (1094)	57% (628)		2.60 ± 3.34	2.35 ± 3.12	0.01 ± 0.13	0.24 ± 0.92	720	
Non-local (368)	59% (218)		3.21 ± 4.02	3.11 ± 3.96	<0.01	0.10 ± 0.51	766	

^1^ Comparison of caries prevalence. ^2^ Comparison of the rank of median dmft score.

**Table 2:** Factors associated with dental caries prevalence of the 5-year-old children (*n* = 1462).

Variables (*n*)	Dental Caries Prevalence	*p*-Value (Chi-Square Test)
Have siblings		<0.001 *
Yes (905)	61%	
No (557)	52%	
Family income below median		<0.001 *
Yes (691)	63%	
No (771)	53%	
Caregiver education below tertiary level		<0.001 *
Yes (708)	63%	
No (754)	53%	
Breast Feeding (first 6 months)		0.046
Yes (1296)	57%	
No (166)	65%	
Snacking before bed		0.013 *
Yes (957)	60%	
No (505)	53%	
Sugary drink at least twice daily		0.004 *
Yes (309)	65%	
No (1153)	56%	
No brushing before age 2		<0.001 *
Yes (501)	65%	
No (961)	54%	
Brushing less than twice daily		0.129
Yes (708)	60%	
No (754)	56%	
No assistance in brushing		0.001 *
Yes (861)	61%	
No (601)	53%	
No use of fluoride toothpaste		0.051
Yes (605)	66%	
No (857)	57%	
Dental visit experience		<0.001 *
Yes (705)	68%	
No (663)	48%	

* Significant difference.

**Table 3:** Factors associated with dental caries severity of the 5-year-old children (*n* = 1462).

Variables (*n*)	dmft Mean ± SD	Rank of Median dmft Score	*p*-Value (Mann–Whitney *U*-Test)
Have siblings			<0.001 *
Yes (905)	3.02 ± 3.70	762	
No (557)	2.32 ± 3.20	682	
Family income below median			0.092
Yes (691)	2.83 ± 3.58	750	
No (771)	2.69 ± 3.48	715	
Caregiver education below tertiary level			<0.001 ^*^
Yes (708)	3.15 ± 3.74	778	
No (754)	2.39 ± 3.28	688	
Breast Feeding (first 6 months)			0.065
Yes (1296)	2.70 ± 3.51	725	
No (166)	3.16 ± 3.68	786	
Snacking before bed			<0.001 *
Yes (957)	3.02 ± 3.74	757	
No (505)	2.26 ± 3.02	683	
Sugary drink at least twice daily			0.002 *
Yes (309)	3.26 ± 3.69	795	
No (1153)	2.62 ± 3.47	715	
No brushing before age 2			<0.001 *
Yes (501)	2.47 ± 3.34	797	
No (961)	3.30 ± 3.81	697	
Brushing less than twice daily			0.129
Yes (708)	2.88 ± 3.59	748	
No (754)	2.64 ± 3.47	716	
No assistance in brushing			<0.001 *
Yes (861)	3.06 ± 3.70	766	
No (601)	2.32 ± 3.23	681	
No use of fluoride toothpaste			0.037 *
Yes (605)	2.93 ± 3.58	758	
No (857)	2.63 ± 3.49	713	
Dental visit experience			<0.001 *
Yes (705)	3.49 ± 3.79	765	
No (663)	2.12 ± 3.21	599	

* Significant difference.

**Table 4:** Zero-inflated portion of regression model of variables and caries-free status (*n* = 1462).

Variables	OR	*p*-Value	95% C.I.
Have siblings			
Yes *			
No	1.479	0.006	1.121–1.951
Family income below median			
Yes *			
No	1.922	<0.001	1.444–2.560
Sugary drink at least twice daily			
Yes *			
No	1.662	0.005	1.163–2.374
No assistance in brushing			
Yes *			
No	1.375	0.027	1.037–1.823
No use of fluoride toothpaste			
Yes *			
No	1.469	0.008	1.104–1.955
Dental visit experience			
Yes *			
No	2.843	<0.001	2.146–3.767

* Reference group.

**Table 5:** Negative binomial portion of regression model of variables and caries severity (*n* = 1462).

Variables	IRR	*p*-Value	95% C.I.
Family income below median			
Yes *			
No	0.766	<0.001	0.673–0.872
Non-local residency			
Yes *			
No	0.741	<0.001	0.639–0.859
Caregiver education below tertiary level			
Yes *			
No	0.853	0.012	0.754–0.965
Snacking before bed			
Yes *			
No	0.830	0.003	0.735–0.936
No brushing before age 2			
Yes *			
No	0.877	0.027	0.780–0.985
Dental visit experience			
Yes *			
No	0.790	<0.001	0.698–0.894

* Reference group.

## Data Availability

The results of the dental examination of each participating child will be shared with the parents or legal guardians via oral health reports. The team will share the results of the study with academia via publications and presentations. The datasets generated in this study will be available from the primary investigator on a legitimate request.

## Supplementary Materials

The following supporting information can be downloaded at: https://www.mdpi.com/article/10.3390/dj13120552/s1, Figure S1: Distribution of dental caries according to tooth position; Table S1: STROBE.

## Abbreviations

The following abbreviations are used in this manuscript:

ECC	Early Childhood Caries
dmft	decayed, missing, and filled primary teeth
dt	decayed primary teeth
mt	missing primary teeth
ft	filled primary teeth
WHO	World Health Organization
CPI	Community Periodontal Index
LED	Light-Emitting Diode
STROBE	STrengthening the Reporting of OBservational studies in Epidemiology
IRB	Institutional Review Board
CI	Confidence Interval
RMB	Renminbi
ZINB	Zero-Inflated Negative Binomial
OR	Odds Ratio
IRR	Incidence Rate Ratio
