# Supplementary figures and images for: Early Childhood Caries and Its Associated Factors Among 5-Year-Old Children in Shenzhen City, China: A Cross-Sectional Study
Source: Dent J (Basel). 2025 Nov 24;13(12):552. doi: 10.3390/dj13120552 (PMC12731665; doi:10.3390/dj13120552)

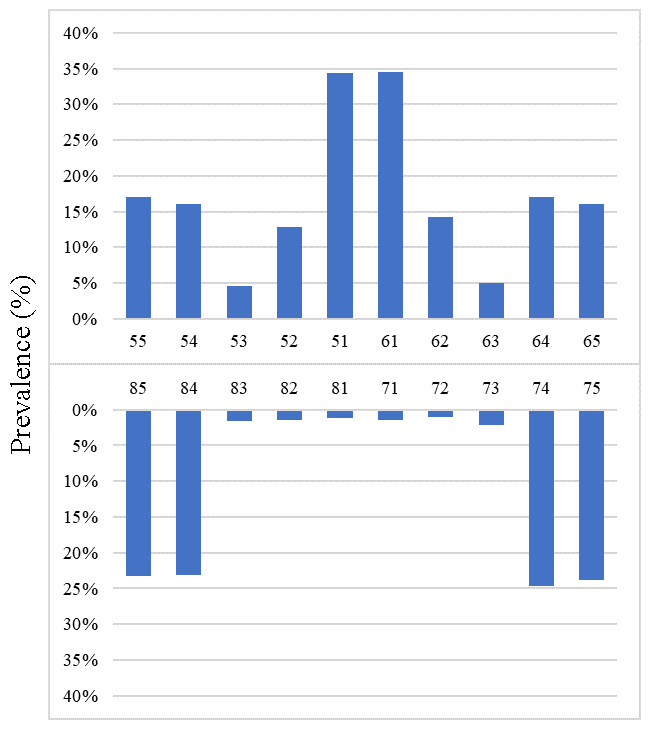

Supplement: Supplementary file 1 [file dentistry-13-00552-s001.zip › Figure S1 Distribution of dental caries according to tooth position.png]
